# Extracellular Vesicles: An Important Biomarker in Recurrent Pregnancy Loss?

**DOI:** 10.3390/jcm10122549

**Published:** 2021-06-09

**Authors:** Nina Rajaratnam, Nadja E. Ditlevsen, Jenni K. Sloth, Rikke Bæk, Malene M. Jørgensen, Ole B. Christiansen

**Affiliations:** 1 Department of Obstetrics and Gynecology, Aalborg University Hospital, 9000 Aalborg, Denmark; n.ditlevsen@rn.dk (N.E.D.); olbc@rn.dk (O.B.C.); 2 Department of Clinical Immunology, Aalborg University Hospital, 9000 Aalborg, Denmark; jennisloth@gmail.com (J.K.S.); rikke.baek@rn.dk (R.B.); maljoe@rn.dk (M.M.J.); 3 Department of Clincial Medicine, Aalborg University, 9000 Aalborg, Denmark; 4 Clinical Institute, Aalborg University, 9000 Aalborg, Denmark

**Keywords:** recurrent miscarriage, recurrent pregnancy loss, habitual abortion, extracellular vesicles, exosomes, microvesicles, EV Array

## Abstract

Recurrent pregnancy loss (RPL) has an estimated incidence of 1–3% of all couples. The etiology is considered to be multifactorial. Extracellular vesicles (EVs) take part in numerous different physiological processes and their contents show the originating cell and pathophysiological states in different diseases. In pregnancy disorders, changes can be seen in the composition, bioactivity and concentration of placental and non-placental EVs. RPL patients have an increased risk of pregnancy complications. The aim of this prospective study was to examine whether measuring different specific EV markers in plasma before and during pregnancy could be used as predictors of pregnancy loss (PL) in women with RPL. Thirty-one RPL patients were included in this study; 25 had a live birth (LB group) and six had a new PL (PL group). Five blood samples were obtained, one before achieved pregnancy and the others in gestational week 6, 8, 10 and 16. Moreover, some of the patients received intravenous immunoglobulin (IVIG) infusions as part of treatment, and it was also examined whether this treatment influenced the EV levels. Seventeen EV markers specific for the immune system, coagulation, placenta and hypoxia were analyzed in the samples with EV Array, a method able to capture small EVs by using an antibody panel targeting membrane proteins. Comparing the LB and PL groups, one EV marker, CD9, showed a significant increase from before pregnancy to gestational week 6 in the PL group. The changes in the other 16 markers were nonsignificant. One case of late-onset PL showed steeply increasing levels, with sudden decrease after gestational week 10 in nine of 17 markers. Moreover, there was an overall increase of all 17 markers after IVIG treatment in the LB group, which was significant in 15 of the markers. Whether increases in EVs positive for CD9 characterize RPL patients who subsequently miscarry should be investigated in future larger studies.

## 1. Introduction

Pregnancy loss (PL) is the most common pregnancy complication which affects approximately 15% of all apparently normal couples [1]. Recurrent pregnancy loss (RPL) is traditionally defined as three or more consecutive pregnancy losses before gestational week 12, although some international guidelines only require two pregnancy losses for the diagnosis [2]. RPL is estimated to have an incidence of 1–3% of all couples [1,3].

The etiology of RPL is considered to be multifactorial and despite thorough investigation, no documented causes for RPL can be found in the majority of cases [4]. Therefore, RPL is often frustrating as evidence-based treatment strategies are limited. 

Several disorders have been connected to RPL, and known risk factors include chromosomal and genetic abnormalities, endocrinological, thrombophilic, autoimmune and alloimmune disorders, and uterine abnormalities [5]. Mechanisms suggested to be involved in the pathogenesis of RPL include insufficient trophoblast invasion, vilitis, and microthrombi in the placental vessels [6]. RPL patients have an increased risk of pregnancy complications in the next pregnancies compared to the background population [7]. Biomarkers in RPL patients that can predict a new PL are probably causally involved in RPL and will be very informative for understanding the syndrome.

There has been an increasing interest over the last years in the diagnostic potential of extracellular vesicles (EV) and their role in different pathological processes. EVs are small plasma membrane vesicles participating in intercellular communication. EVs take part in numerous different processes e.g., relating to the immune system. Inside the vesicles, a cargo is contained and protected. The cargo provides information about the originating cell and pathophysiological states, e.g., in autoimmune diseases and cancer [8]. EVs can be classified by their biogenetic pathway, physical characteristics, and composition. There are three main subgroups of EV: apoptotic bodies, microvesicles, and exosomes. This division is based on the biogenetic mechanism of formation and release and size [8].

In pregnant women, the EV cargo increases compared to nonpregnant individuals, and might thereby contribute to modulation of the maternal immune response against the fetus [9,10]. The placenta is the main source of EVs during pregnancy and releases exosomes into the maternal circulation from as early as the 6th week of gestation [11]. The placenta transfers genetic information to target cells to regulate the feto-maternal metabolic homeostasis, tolerance of the immune system through inhibition of maternal T-cells, and regulation of angiogenesis and endothelial cell migration [12]. 

In pregnancy disorders, changes can be seen in the composition, bioactivity, and concentration of placental and non-placental EVs. Specifically, increased secretion has been described in gestational diabetes and preeclampsia. Placental exosomes possibly take part in pathological processes of pregnancy, like preterm birth, and premature rupture of membranes [12]. EVs may have a significant role in modulating maternal immunity in order to achieve a successful pregnancy, representing many immunological processes by elimination of pathogens without harming the fetus [13,14,15,16]. However, regarding placenta-derived EVs it is still unknown whether the changes in bioactivity is a result of cargo-alteration, whether negative effects in pathological pregnancies is caused by the altered cargo and lastly whether the effect is a possible combination of increased EVs and their cargo. Thus, it could be the changes in quantity and/or quality of EVs [17]. 

The purpose of this prospective study was to investigate whether measuring plasma levels of EVs with different surface markers specific for immunological processes, coagulation, placenta, and hypoxia before and during pregnancy can be used as predictors for new PL in RPL patients.

## 2. Materials and Methods

The prospective cohort study was performed at a tertiary university-affiliated center for RPL from the period July 2018–July 2019. This study period was defined in the protocol approved by the ethics committee and was restricted by the grants for two of the researchers (NR and NED). In the protocol, a sample size calculation was made, based on data from our pilot study [18]. It was estimated that 42 patients who obtained pregnancy should be included to be able to assess whether patients with EVs that increased strongly in early pregnancy had a 47% higher miscarriage rate compared with patients with stable levels of EVs, which was an observation from our pilot study [18]. In the study period, 58 consecutively referred suitable RPL patients gave oral and written informed consent for participation and had blood samples taken before pregnancy. However, unfortunately only 31 patients achieved pregnancy in the study period and had at least two blood sample taken in pregnancy and were thus informative. 

### 2.1. Study Population

Inclusion criterias: (1) RPL defined as three or more consecutive pregnancy losses (confirmed miscarriages and biochemical pregnancies), (2) age ≥18 years, (3) achievement of pregnancy within the study period, and (4) consecutively enrolled.

Exclusion criterias: (1) significant uterine abnormalities, (2) significant chromosomal aberrations in the couple, and (3) not able to understand Danish.

### 2.2. Treatment and Blood Withdrawal 

Among the 31 included patients, 12 patients received intravenous immunoglobulin (IVIG) starting before conception and continued every week and later every second week until pregnancy week 12–14. The decision to treat with IVIG (Privigen from CSL Behring 100 mg/mL, 25–35 g at each infusion) was mainly based on a high number (at least four) of previous miscarriages or a history of second-trimester miscarriages.

Blood samples were collected as one baseline sample before pregnancy (and first IVIG infusion) and throughout the patient’s first subsequent pregnancy at gestational week 6, 8, 10, and 16. If the patient was treated with IVIG, blood samples were taken before the infusion was provided.

Venous peripheral blood was obtained using 3 × 2.7 mL citrate tubes. The samples were centrifuged afterwards at room temperature (RT), 4000× *g* for 10 min, always within 3 h of withdrawal. Afterwards, the plasma was removed, aliquoted, and stored at −80 ℃ until further EV array analysis.

### 2.3. EV Array

The extracellular vesicle array (EV Array) is a sandwich ELISA-based method optimized to capture smaller types of EVs (sEV), such as exosomes and exosome-like vesicles with a diameter up to ∼150 nm. The capturing of sEVs is performed with the use of an antibody panel targeting selected membrane- or membrane-associated proteins [19]. The EV Array constitutes a fast, automated, economical and highly sensitive method for exploration of plasma-sEVs carrying CD9, CD63 and/or CD81. EV Array analysis was performed on plasma samples from the 31 RPL patients with 17 different EV surface markers (Table 1).

#### 2.3.1. Antibodies for Production of the EV Array

The general markers CD9, CD63 and CD81 were chosen as they mentioned earlier are used in the EV Array analysis. Annexin V, another general EV marker, has been found with increased levels in RPL patients [20]. 

Several disorders have been connected to RPL and known risk factors are among other immunological and trombophilic disorders [5]. Various immunological markers (CD4, CD9, CD45, HLA DRP/DQ/DR, HLA G, FasL, TRAIL and Hsp70) were chosen. A study found both CD9+ T-cells to be significantly higher and the CD4/CD8 ratio decreased in RPL patients compared to controls [21]. Several studies have also found higher levels of activated T-cells in peripheral blood of RPL and infertile patients [22,23,24]. HLA molecules play an important role in the adaptive immune system. Investigation of HLA polymorphisms is used in some clinics as a diagnostic test in RPL patients [3]. One study suggested that FasL- and TRAIL-carrying exosomes, which are able to convey apoptosis, are secreted by the placenta and can be tied up to the immunomodulatory and protective role of the human placenta to its exosome-secreting ability [25]. Lastly, of the immunological markers, Hsp60/70 equal to or more than 6 until gestational week 12 has been reported to be associated with high likelihood for miscarriage [26].

The coagulation markers CD31 and CD142 have also been studied in RPL patients, two studies [27,28] found endothelial microparticles significantly increased compared to controls, whereas another study [29] found significant decreases of CD31+ and CD41- in RPL patients compared to controls. 

PLAP is specific to the placenta, one study proposed that polymorphisms in PLAP could be associated with in vitro fertilization success and RPL [30]. CAIX/CAXII are hypoxia markers belonging to the carbonic anhydrase family consisting of enzymes that play an important role in pH regulation. CAIX has to our knowledge not been thoroughly explored in relation to pregnancy or miscarriage.

#### 2.3.2. EV Array Analysis

Microfluor2 plates (Thermo Scientific, Waltham, MA, USA) were customized by printing antibodies into microsized spots using a SciFLEXARRAYER S12 microarray printer (Scienion AG, Dortmund, Germany). All antibodies were printed in triplicates and for quality control, each sample was analyzed three times and the mean of these was used for analysis. For positive controls, biotinylated human IgG containing 5% trehalose was used and for negative controls PBS with 5% trehalose was used.

The analysis was performed as follows. First, the plates were blocked with 50 mM ethanolamine, 0.1 M Tris, 0.1% SDS, pH 9.0, and subsequently a washing procedure was performed with wash buffer (0.2% Tween^®^ 20 in PBS) using a plate washer (TECAN Group Ltd., Männedorf, Switzerland). Afterward, a 50 µL sample was added to each well and diluted in wash buffer to a total volume of 100 µL followed by incubation for two hours at RT. After the initial incubation, the plates were placed to incubate overnight at 4 °C. The following day the plates underwent another washing procedure prior to incubation with a mixture of biotinylated detection antibodies (antihuman-CD9, -CD63 and -CD81, LifeSpan BioSciences, Seattle, WA, USA) diluted in wash buffer 1:1.500 for two hours at RT. Afterwards, plates were washed again before adding streptavidin-Cy3 (Life Technologies, Carlsbad, CA, USA) diluted 1:1.500 in wash buffer that was carried out for detection of the amount of captured EVs. After a 30 min incubation, the plates were washed first in wash buffer and, finally, in deionized water and lastly dried.

Scanning of the plates and spot detection of the antibody markers by fluorescence readout was performed using a sciREADER FL2 scanner (Scienieon AG, DE). Image analysis and calculation of total fluorescence intensity were performed using the sciREADER FL2 software.

### 2.4. Statistical Analysis 

Population characteristics were summarized in Table 2 with mean and standard deviation (SD) for continuous variables and counts for discrete variables.

For each of the 17 antibody markers, a linear mixed model was used to estimate the progression throughout gestation for the LB group. The estimated mean progression and confidence interval was displayed for graphical illustration. The observed progression for the same marker in the PL group was added to the plot to illustrate divergence from the LB mean. The changes in antibody markers from baseline to in gestational week 6 was compared for the PL and LB group using Kruskal–Wallis rang-test. 

Within each group (PL and LB) some of the patients received IVIG treatment before and during pregnancy. To examine the influence of the IVIG treatment on the levels of the 17 EV markers for the LB group, graphs divided by IVIG treatment (IVIG/no IVIG) were made to visualize individual progression during pregnancy. Further, for each of the 17 EV markers, the changes from baseline to sixth gestational week was computed for IVIG treatment using a *t*-test. A confidence interval for the mean difference in changes from baseline to the sixth gestational for IVIG/no IVIG was presented together with the *t*-test for no difference between IVIG/no IVIG. 

Graphs and statistical analysis were drawn and performed in the statistical program R, version 3.6.3. Any *p*-value less than 0.05 was considered as significant in this study.

## 3. Results

### 3.1. Baseline Characteristics

A total of 31 RPL patients were included in this study. This group was divided into subsequent PL (*n* = 6) and LB (*n* = 25) groups. Within the PL group, four received IVIG treatment and 10 in the LB group received IVIG. The characteristics of all the women including age, BMI, primary/secondary RPL, number of previous PL, IVIG treatment and gestational week of LB are presented in Table 2.

### 3.2. Pregnancy Loss vs. Live Birth

**Figure 1:**
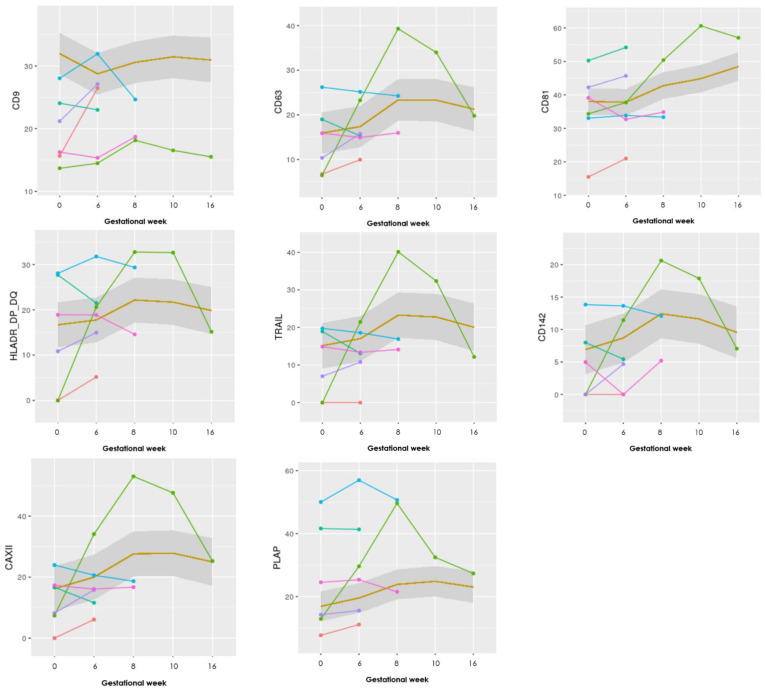
Graphs showing the progression of eight EV markers examined in the EV Array. The LB group (*n* = 25) is illustrated as a mean within the grey box, whereas the PL group (*n* = 6) is individually visualized with six different colors. Gestational week is depicted on the *x*-axis and absolute levels of the marker along the *y*-axis (the remaining nine markers can be seen in Appendix B).

Seventeen different antibodies were printed in triplicates and used to capture and analyze the content of the sEVs in 50 μL of unpurified plasma. The markers to include in this study were selected from the literature described under Table 1. To assess any difference in sEV levels during pregnancy between the two groups (LB and PL), graphs for all 17 antibody markers were made, and eight specifically selected (Figure 1). These illustrate the absolute levels of each EV marker chosen for this study for each of the six patients in the PL group, and a mean for the LB group. 

Looking at the marker values for the progression in the pregnancies ending in PL, they show overall lower levels of the general EV marker CD9 (in five of six patients with PL compared with the mean of the LB group), whereas the other EV markers Annexin V, CD63, and CD81 showed more similar levels compared to the mean of the LB cases.

Specifically, one case with a late PL in gestational week 20 marked with green (Figure 1), shows steeply increasing levels, with a sudden decrease after gestational week 10 in 9 of the 17 markers (CD63, HLA-G, HLA-DR/DP/DQ, TRAIL, Hsp70, CD142, CAIX, CAXII, and PLAP—see Appendix B for the other graphs).

In the PL group, only one or two blood samples were obtained after achieved pregnancy due to early PLs, besides the one case of late PL. Therefore, to examine whether there was any significant difference in the progression of the different markers, the median differences between the samples taken in gestational week 6 compared to the baseline sample taken before pregnancy were calculated for all the included patients’ samples. A range test between the two groups was performed to assess any significant difference (Table 3). 

CD9 showed a statistically significant difference between the PL and LB group. Differences in the progression of the other markers were nonsignificant.

### 3.3. Intravenous Immunoglobulin

**Figure 2:**
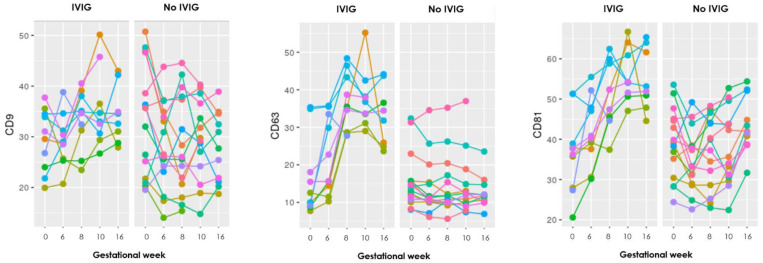

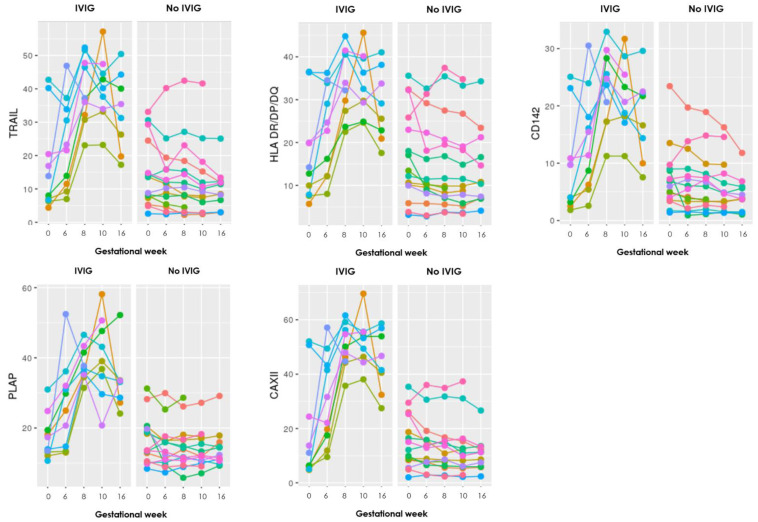
Graphs showing the progression during pregnancy of eight EV markers examined with the EV array in the LB group, where each color represents an individual person. To the left are those who received IVIG treatment and to the right those who did not. Gestational week is depicted on the *x*-axis and absolute levels of the marker along the *y*-axis (the remaining nine markers can be seen in Appendix B).

Twelve of the included RPL patients received IVIG as part of treatment, four from the PL group and ten from the LB group. To examine whether IVIG had any effect on the sEV levels, firstly graphs for each of the 17 markers were made to visualize the progression during pregnancy. To exempt any influence of PL and to visualize the progression until week 16 (typically after five to six IVIG infusions), the graphs only included the LB group (Figure 2). 

The graphs show distinct steep increasing levels of sEV carrying almost all the EV markers in those who received IVIG treatment, compared to the mostly stable sEV levels throughout gestation in those who did not receive IVIG treatment.

To evaluate whether these visually clear differences between those who received IVIG treatment or not were significant, a *t*-test was performed for the difference between each of the samples taken at the baseline before pregnancy and first IVIG infusion and in gestational week 6 (Table 4).

In the LB group, one extra sample was taken in three patients 14 days after the first IVIG infusion and before pregnancy, to see whether IVIG on its own could influence the EV levels. All 17 markers showed increasing EV levels after the IVIG infusion. 

## 4. Discussion

Pregnancy complications are hypothesized to have their origin in disorders of early placentation [11]. If these changes affect the placenta this would arguably affect EV production during early pregnancy. Women at risk of developing pregnancy complications such as RPL patients might benefit from identification of changes in EV secretion and thereby provide an opportunity to develop clinically useful early pregnancy screening biomarkers. 

To determine the levels and phenotypes of sEVs in plasma from patients with RPL, the established protein microarray-based analysis EV array was used. The EV array is optimized to catch and detect the smaller types of EVs, such as exosomes and exosome-like vesicles, with diameters up to ∼150 nm [18].

We measured the level of sEVs with relevant surface markers in consecutive blood samples taken before and during pregnancy in women with RPL, some of them treated with IVIG. Levels of sEVs or changes in concentration in early pregnancy were correlated with subsequent pregnancy outcomes.

The detection of sEV was performed using antibodies against the markers CD9, CD63 and CD81 as not all sEVs necessary express equal amounts of these tetraspanins and that expression depends on their origin [31], which is in accordance with the MISEV2018 (Minimal Information for Studies of Extracellular Vesicles) guidelines [32]. The EV array has previously been shown to capture and detect sEVs present in a sample [18], which is why it was chosen not to perform additional experiment to verify the presence of sEVs.

In this study, the levels of sEVs with one marker, CD9, was significantly different between the PL and LB group when comparing the EV levels measured at baseline and gestational week 6. CD9 plays a relevant role in exosome genesis, which is why it is used as an EV marker. The other markers AnnexinV, CD81, and CD63, often used as general EV markers, did not show any significant changes between the groups. However, it has been shown that some of the tetraspanins are not specific for all EV types and can be detected on other cell-types as well [33].

Therefore, it is difficult to conclude what importance the change in CD9 positive sEVs has. When comparing this with the graphs (Figure 1), the levels of CD9 increase from sample 1 to 2, in three of the six patients in the PL group, whereas the mean CD9 level for the LB group in the same period shows a decrease. Whether increases in sEVs positive for CD9 characterize RPL patients who subsequently miscarry should be investigated in future larger studies.

In previous studies, it has been reported that the level of circulating EVs increases in pregnancy and that they progressively increase with gestational age, with the highest concentration in the third trimester [34,35,36]. This does not correlate with the findings in our study where an initial increase in levels of sEVs is seen until gestational week 8–10 in the LB group but afterwards, the levels are stagnating or slightly declining until week 16. However, it must be borne in mind that many patients received IVIG infusions until week 14, which may confound the results.

Visually the graphs for the progression of the 17 different EV markers showed that most of the PL group had a similar course compared to each other throughout pregnancy, although the baseline values could differ. Interestingly, one patient with a late-onset intrauterine death of a normal, but severely growth-retarded fetus in week 20 showed a clearly different pattern of progression in at least nine of the 17 markers (CD63, HLA-G, HLA-DR/DP/DQ, TRAIL, Hsp70, CD142, CAIX, CAXII, and PLAP) in Figure 1. There was a steep increase in EVs positive for these markers until gestational week 8–10 and then a steep decline in the levels in gestational week 16, at a time where ultrasound examination revealed a live fetus with normal biometries.

It could be speculated, that some pathological processes had begun in the placenta around week 8–10 releasing EVs with the mentioned markers in great quantities into the maternal circulation. In week 16, the pathological processes had burned out leaving a damaged placenta but an apparently normal fetus still living for some weeks. 

The markers of interest in the aforementioned case include CD63, immunological markers HLA-G, HLA-DR/DP/DQ, TRAIL, Hsp70, CD142, CAIX, CAXII, and lastly PLAP. The involvement of the markers related to immune function and hypoxia may suggest that some immune activation associated with the release of hypoxic cells released from the placenta took place between week 8–10 but manifested itself only in gestational week 20. Clarification of this phenomenon in further studies will bring us closer to the understanding of the background for many cases of unexplained late intrauterine fetal deaths.

The immunological phases of pregnancy have been described as the first/early second trimester, with active implantation and placentation, being characterized by a pro-inflammatory environment, the second/early third trimester as an immunosuppressive stage to uphold maternal-fetal tolerance, and lastly the last part of the third trimester with a revival of proinflammatory environment for expulsion of fetus and placenta [15]. 

During pregnancy, the levels of sEVs are greater than medium/large EVs and are considered anti-inflammatory [13]. Most studies have suggested an immunosuppressive role of sEVs, but some have reported proinflammatory functions [37]. Only sEVs are measured with the EV Array [18]. A review [38] looked at both platelet-derived (PMV), endothelial-derived (EMV) and leukocyte-derived microvesicles (LMV) roles in inflammation and inflammatory-related disorders. PMVs show mostly proinflammatory properties but can combine these actions with the ability to reduce inflammation. For the EMVs they may be qualified biomarkers of endothelial dysfunction in different infections. The LMVs primary function is activation of proinflammatory response in other cell types and are seen increased in infectious and inflammatory diseases.

Regarding IVIG treatment, a statistically significant higher increase of sEVs was found in the IVIG treated RPL patients compared with no IVIG treated patients. This was seen for 12 of the 17 EV markers, and moreover the other five also showed increases, just not significant. These findings suggest that treatment with IVIG contributes to increased concentration of sEVs in the plasma of pregnant RPL patients. Another study by Jørgensen et al. [17] also found a significant increase of sEVs in RPL patients after receiving IVIG compared to placebo. Moreover, sEV levels were measured in three LB patients, 14 days after their first IVIG infusion (before pregnancy), and it showed increasing levels of all 17 markers, which shows that IVIG probably has a specific effect on the sEV levels outside of just pregnancy.

This phenomenon might be based on a systemic effect as IVIG has several immunomodulatory effects involving cytokines, the complement system, and inflammatory cells, and one of the ways inflammatory cells exert immunomodulation is by secretion of exosomes [39]. 

EVs potential as diagnostic biomarkers have been investigated for diseases such as systemic lupus erythematosus [40] and different cancer types such as prostate [41] and ovarian [42] cancer. The studies regarding RPL patients and microparticles have made analyses by different methods such as flow cytometry [28,29,43], which is more expensive as it is time- and sample-consuming. It can therefore be difficult to compare the findings of this study as the use of nonstandardized methods may compromise comparative analysis. One study used an ELISA-based method and CD63 as an EV marker looking at women with healthy pregnancies or PL. In this study, no significant overall difference in EV levels was found, but a significant increase in plasminogen activator in spontaneous PL [44]. 

The EV Array used in our study is faster, more economical, highly sensitive, and only requires a small amount of sample material [18], all useful criteria for high diagnostic value in the clinic. EV Array is fluorescent-based and provides semiquantitative results. This was considered in the current study as each patient acted as their own control from the baseline sample compared to the following blood samples. 

Because of the limited size of the study population, (unfortunately only 31 of the planned 42 patients were included), it is difficult to conclude on any predictive value of the EV markers analyzed. In the future, a larger study population would benefit the statistical analysis, and moreover, a healthy pregnant control group could help to give insight to normal changes in EVs during pregnancy. There is also potential in looking closer at different pregnancy complications, which are more frequently seen in RPL patients, such as gestational diabetes, preeclampsia, intrauterine growth retardation, and preterm birth.

## 5. Conclusions

The results of this study show an overall increase of all 17 EV markers after IVIG treatment in pregnant RPL patients, and 15 markers with a significant increase. Moreover, CD9 was the only marker showing a significant difference between the LB and PL groups. Whether increases in sEVs positive for CD9 characterize RPL patients who subsequently miscarry should be investigated in future larger studies.

## Figures and Tables

**Table 1:** Overview of the 17 EV markers used in this study (see Appendix A for clone and producent).

Group	Full Name	Abbreviation
**General EV markers**	Annexin V	AnnexinV
	Cluster of Differentiation 9	CD9
	Cluster of Differentiation 63	CD63
	Cluster of Differentiation 81	CD81
**Immune/haematological markers**	Cluster of Differentiation 4	CD4
	Cluster of Differentiation 8	CD8
	Cluster of Differentiation 45	CD45
	Major histocompatibility complex, class I, DR, DP, DQ	HLA DR/DP/DQ
	Major histocompatibility complex, class I, G	HLA-G
	Fas ligand	FasL
	TNF-related apoptosis-inducing ligand	TRAIL
	Heat-shock protein 70	Hsp70
**Coagulation markers**	Cluster of Differentiation 31/platelet endothelial cell adhesion molecule	CD31
	Cluster of Differentiation 142/tissue factor	CD142
**Placental/hormonal markers**	Placental alkaline phosphatase	PLAP
**Hypoxia markers**	Carbonic anhydrase IX	CAIX
	Carbonic anhydrase XII	CAIXII

**Table 2:** General characteristics of the study population.

	PL (*n* = 6)	LB (*n* = 25)
Age, (y, mean ± SD)	38 ± 3	32 ± 4
BMI, (kg/m^2^, mean ± SD)	28 ± 4	24 ± 6
Primary RPL	5	17
Secondary RPL	1	8
Number of previous PL ≤ 3	0	13
Number of previous PL = 4	2	12
Number of previous PL ≥ 5	4	0
IVIG treatment	4	10
Gestational week at birth		
≥37		24
<37		1

**Table 3:** Shows the mean (SD) for the difference between baseline and sixth gestational week measurement for each marker in the two groups PL and LB, and *p*-values.

EV Markers	PL (*n* = 6)	LB (*n* = 25)	*p*
AnnexinV	−5.1 (5.2)	−5.1 (14.8)	NS
CD9	−3.2 (4.6)	3.2 (7.6)	0.04
CD63	−3.3 (7.4)	−1.4 (7)	NS
CD81	−1.8 (4.3)	0.2 (8.8)	NS
CD4	0.9 (2)	0.2 (5.5)	NS
CD8	−1 (4.5)	−0.5 (2.9)	NS
CD45	−4.4 (9.6)	−1.9 (9.9)	NS
TRAIL	−2.8 (9.6)	−1.8 (9.6)	NS
HLA DR/DP/DQ	−4.6 (8.9)	−1.1 (7.3)	NS
HLA G	−2.6 (5.1)	−0.4 (5.7)	NS
FasLigand	−4 (9.3)	−5.7 (16.2)	NS
Hsp70	0.1 (5.2)	−1.5 (5.6)	NS
CD31	−4.2 (5.9)	−3.6 (17)	NS
CD142	−1.4 (5.9)	−1.8 (6.5)	NS
PLAP	−4.8 (6.4)	−2.6 (9.8)	NS
CAIX	−2.4 (9.9)	−3.7 (7.1)	NS
CAIXII	−5.1 (11.7)	−3.8 (13.2)	NS

NS = nonsignificant.

**Table 4:** Mean difference of IVIG in changes in progression of sEV levels measured at baseline (S1) to sixth gestational week (S2).

EV Markers	CI (95 %) Mean Difference (IVIG/no IVIG) of Changes (S1–S2)	*p*
AnnexinV	−30.29; −3.33	0.019
CD9	−11; 0.92	NS
CD63	−14.43; −1.74	0.017
CD81	−16.16; −3.27	0.005
CD4	−9.24; 1.18	NS
CD8	−5.42; −0.27	0.033
CD45	−19.37; −0.67	0.038
TRAIL	−17.53; 0.62	NS
HLA DR/DP/DQ	−14.28; −1.96	0.014
HLA G	−8.93; 2.09	NS
FasL	−33.33; −3.47	0.021
Hsp70	−9.14; 1.07	NS
CD31	−34.78; −4.55	0.016
CD142	−10.4; 0.321	0.063
PLAP	−20.02; −2.62	0.016
CAIX	−13.62; −2.69	0.006
CAIXII	−26.49; −1.56	0.031

NS = nonsignificant.

**Figure A1:**
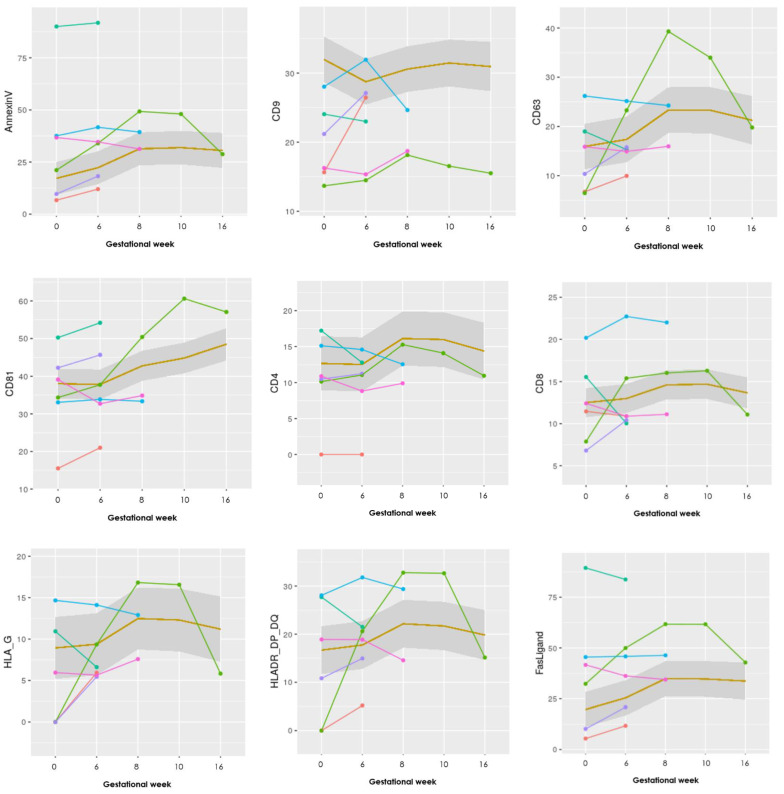

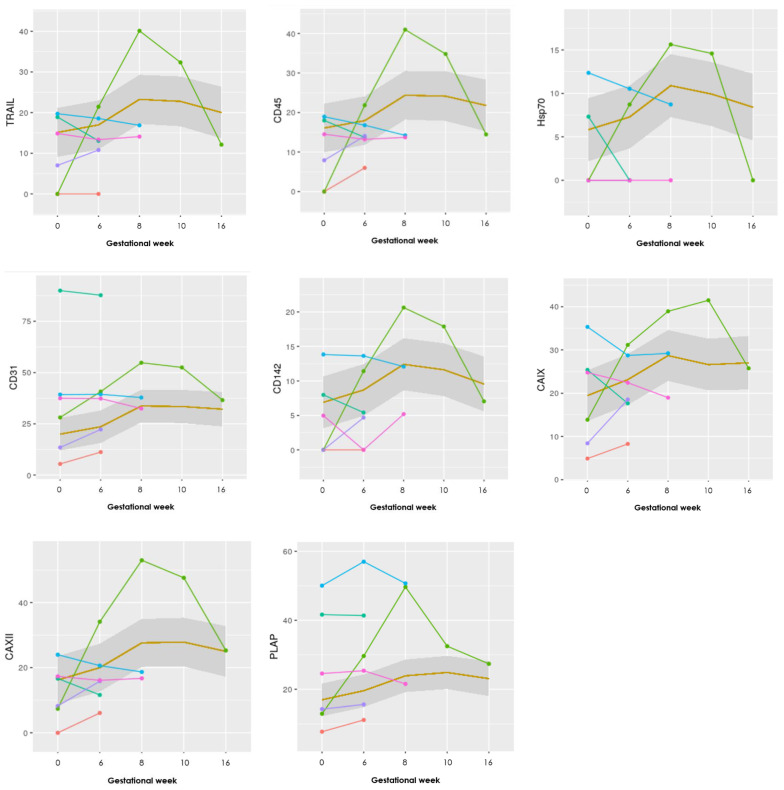
Graphs showing the progression of all 17 EV markers examined in the EV array. The LB group (*n* = 25) is illustrated as a mean within the grey box, whereas the PL group (*n* = 6) is individually visualized with six different colors. Gestational week is depicted on the *x*-axis and absolute levels of the antibody marker along the *y*-axis.

**Figure A2:**
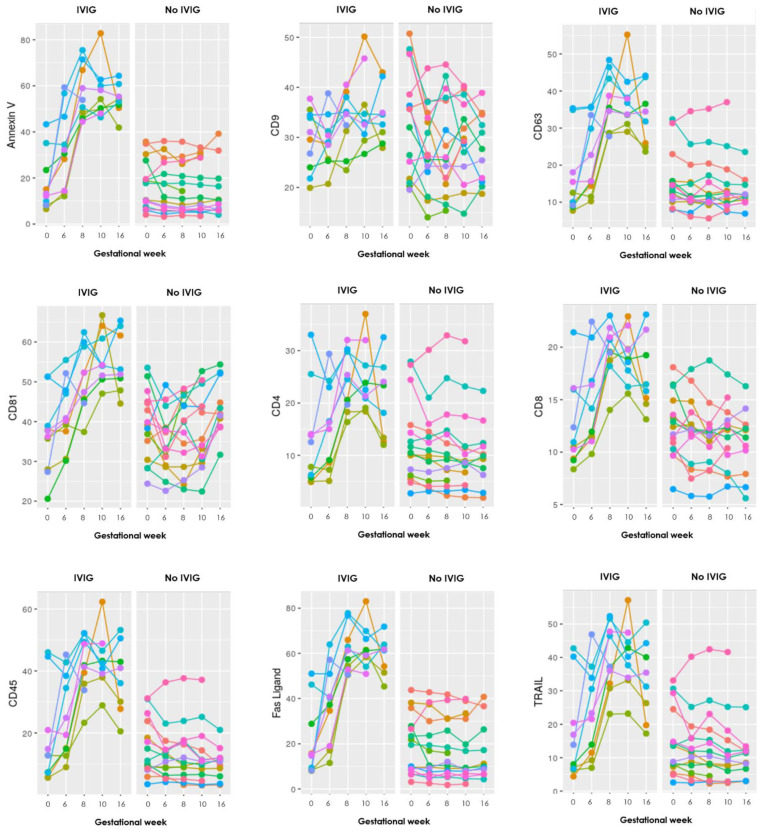

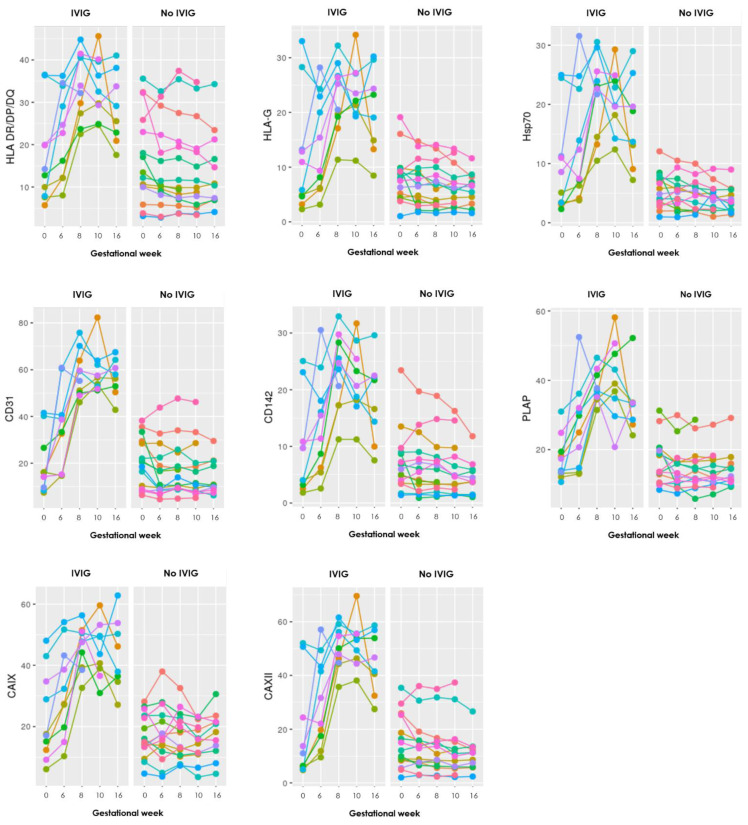
Graphs showing the progression during pregnancy of all 17 EV markers examined with the EV array in the LB group, where each color represents an individual person. To the left are those who received IVIG treatment and to the right those who did not. Gestational week is depicted on the *x*-axis and absolute levels of the antibody marker along the *y*-axis.

## Data Availability

Not applicable.

## Appendix A

*General EV markers*: Annexin V, CD9 (Ancell corporation, MN, USA), CD63 (Bio-Rad, CA, USA), CD81 (Ancell corporation, MN, USA)

*Immune*/*hematological markers*: CD4 (34930), CD8 (37006), CD45 (2D1), HLA DR/DP/DQ (B-145/IVA12; Caprico Biotechnologies, GA, USA), HLA-G (87G, Novus Biologicals, CO, USA), FAS-ligand, TRAIL (75411; R&D Systems, CO, USA), Hsp70 (242707)

*Coagulation markers*: CD31, CD142 (323514)

*Placenta/hormonal markers*: PLAP (8B6, Santa Cruz Biotechnology, TX, USA)

*Hypoxia markers*: CAIX (Abcam, UK), CAXII (315602)
